# Successful production of genome-edited rats by the rGONAD method

**DOI:** 10.1186/s12896-018-0430-5

**Published:** 2018-04-02

**Authors:** Tomoe Kobayashi, Masumi Namba, Takayuki Koyano, Masaki Fukushima, Masahiro Sato, Masato Ohtsuka, Makoto Matsuyama

**Affiliations:** 1 Division of Molecular Genetics, Shigei Medical Research Institute, 2117 Yamada, Minami-ku, Okayama 701-0202 Japan; 2 Shigei Medical Research Hospital, Minami-ku, Okayama 701-0202 Japan; 3 Section of Gene Expression Regulation, Frontier Science Research Center, Kagoshima University, Kagoshima, Kagoshima 890-8544 Japan; 4 Department of Molecular Life Science, Division of Basic Medical Science and Molecular Medicine, Tokai University School of Medicine, Isehara, Kanagawa 259-1193 Japan; 5 Center for Matrix Biology and Medicine, Graduate School of Medicine, Tokai University, Isehara, Kanagawa 259-1193 Japan; 6 The Institute of Medical Sciences, Tokai University, Isehara, Kanagawa 259-1193 Japan

**Keywords:** CRISPR/Cas9, *i*-GONAD, rGONAD, Rat, Knock-out, Knock-in, In vivo electroporation

## Abstract

**Background:**

Recent progress in development of the CRISPR/Cas9 system has been shown to be an efficient gene-editing technology in various organisms. We recently developed a novel method called Genome-editing via Oviductal Nucleic Acids Delivery (GONAD) in mice; a novel in vivo genome editing system that does not require ex vivo handling of embryos, and this technology is newly developed and renamed as “*i*mproved GONAD” (*i*-GONAD). However, this technology has been limited only to mice. Therefore in this study, we challenge to apply this technology to rats.

**Results:**

Here, we determine the most suitable condition for in vivo gene delivery towards rat preimplantation embryos using tetramethylrhodamine-labelled dextran, termed as *R*at improved GONAD (rGONAD). Then, to investigate whether this method is feasible to generate genome-edited rats by delivery of CRISPR/Cas9 components, the tyrosinase (*Tyr*) gene was used as a target. Some pups showed *albino*-colored coat, indicating disruption of wild-type *Tyr* gene allele. Furthermore, we confirm that rGONAD method can be used to introduce genetic changes in rat genome by the ssODN-based knock-in.

**Conclusions:**

We first establish the rGONAD method for generating genome-edited rats. We demonstrate high efficiency of the rGONAD method to produce knock-out and knock-in rats, which will facilitate the production of rat genome engineering experiment. The rGONAD method can also be readily applicable in mammals such as guinea pig, hamster, cow, pig, and other mammals.

**Electronic supplementary material:**

The online version of this article (10.1186/s12896-018-0430-5) contains supplementary material, which is available to authorized users.

## Background

The laboratory rat (*Rattus norvegicus*) has long been used in many studies to model a specified trait of human diseases (as exemplified by hypertension, diabetes and renal diseases), and for testing the drugs [1–4]. For example, Wistar Kyoto (WKY) strain is known to be uniquely susceptible to crescentic glomerulonephritis among the rat strains tested [5, 6]. Injection of isologous monoclonal antibodies caused anti-glomerular basement membrane antibody-induced glomerulonephritis (anti-GBM nephritis) in WKY rats [7, 8]. Notably, there is no mouse strain showing anti-GBM nephritis induced by such types of monoclonal antibodies. However, despite the usefulness/importance of rats as experimental animals, production of genetically engineered rats has yet not been extensively proceeded during the past decades. Gene targeting using mouse ES cells used as an invaluable tool for exploring gene functions in mammals [9], however, these advantages are restricted in limits of the mouse model.

Establishment of successful rat ES cell culture was just made in 2010 [10]. In 2011 to 2013, a more convenient and simpler technology, called genome-editing technology as exemplified by zinc finger nucleases (ZFN) [11, 12], transcription activator-like effector nucleases (TALEN) [13], and CRISPR/Cas9 [14–16] capable of modifying a specific gene function in mammals appeared. With these technologies, it becomes possible to create genetically modified rats in a more convenient manner than ever.

The recent CRISPR/Cas9 system is the simplest for generating animals carrying a modified genome. The system consists of Clustered Regularly Interspaced Short Palindromic Repeats (CRISPR) RNA and CRISPR-associated (Cas) nuclease protein [17, 18]. This system is efficiently generated with knock-out and knock-in animals at targeted sequences [19, 20]. The CRISPR/Cas9 system simplifies the procedure for producing mutant animals, however, genome engineering in rat is still difficult task in most laboratories [21].

The most widely employed procedures for creating genome-edited animals include 3 major ex vivo embryo handling steps [15, 16, 19]: namely 1) isolation of zygotes from a pregnant female that were previously mated with a male, 2) zygote microinjection of genome editing components, and 3) surgical transfer of microinjected zygotes into oviduct of a pseudopregnant female. These 3 steps require very high level of technical expertise and its proficiency of the researchers/technicians to perform these procedures, and expensive apparatus such as micromanipulator.

To simplify these complexed and more laborious processes, Ohtsuka and their colleagues developed a novel genome engineering method, called Genome-editing via Oviductal Nucleic Acids Delivery (GONAD) in mice [22–24]. This method involves in the in vivo genome-editing towards early preimplantation embryos present in an oviduct of pregnant females. Therefore, it does not require above-mentioned ex vivo handling of embryos, such as isolation of zygotes, zygote microinjection and transfer of the injected embryos to recipient females. In our first GONAD attempt using Cas9 mRNA and sgRNA, genome editing efficiency was about 25% [22]. It was subsequently improved up to nearly 100% by using Cas9 protein and crRNA/tracrRNA complex (*i*mproved GONAD; *i*-GONAD) [24]. Although *i*-GONAD method is possibly applied to other organisms, unfortunately, this technology has been limited only to mice.

In this study, we attempted to apply this technology to rats. For this purpose, we used pigmented DA and albino WKY rat strains. We here examined whether 1) CRISPR/Cas9-mediated induction of *indel* is possible towards the wild-type tyrosinase (*Tyr*) locus, and 2) CRISPR/Cas9-mediated knock-in (KI) of single stranded (ss) oligonucleotides (ODN) in the mutated *Tyr* is possible by using the rGONAD method.

## Results

### Determination of optimal electroporation efficiency for rGONAD

**Fig. 1:**
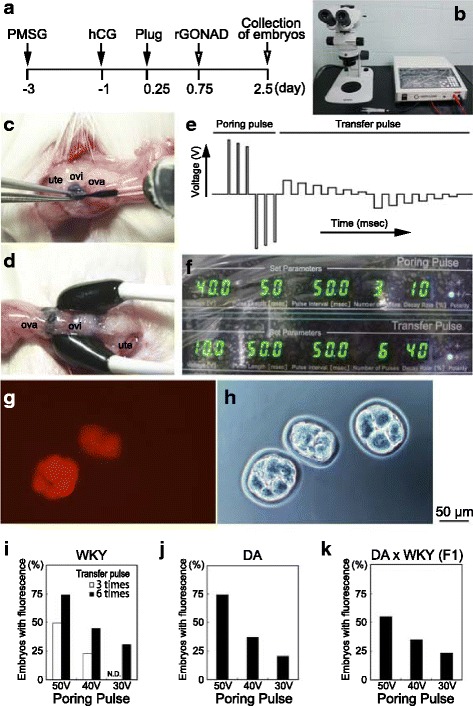
Determination of optimal electroporation efficiency for rGONAD. **a** Scheme of experimental procedures for evaluation of electroporation efficiency using GONAD method. **b** SZX7 stereomicroscope and Super Electroporator NEPA21. **c** Tetramethylrhodamine-labelled dextran is instilled into the oviduct lumen using a micropipette inserted through the oviductal wall near the infundibulum. **d** After the injection, the oviductal regions were covered with a piece of wet paper, and then, electroporation is performed using tweezer-type electrodes. **e**, **f** Scheme (**e**) or diagram (**f**) of electroporation conditions delivered of by an electroporator. This electroporation parameters were; Poring pulse; 40 V, 5 msec pulse, 50 msec pulse interval, number of pulse 3 times, 10% decay (± pulse orientation) and Transfer pulse; 10 V, 50 msec pulse, 50 msec pulse, number of pulse 6 times, 40% decay (± pulse orientation). **g**, **h** Fluorescence analysis of Tetramethylrhodamine-labelled dextran using GONAD method. **i**-**k** Graph shows analysis of the percentage of electroporation efficiency in WKY (**i**), DA (**j**), and DA x WKY (**k**). Ova, ovary; Ovi, Oviduct; Ute, uterus. Scale bars: 50 μm (**g**, **h**)

In our mouse *i*-GONAD method, the electroporation was performed at 0.7 day of pregnancy (E0.7), at which embryonic stage corresponds to late 1-cell mouse embryos [24]. In the present study with rats, it was confirmed that over 80% of embryos collected from females (both with or without super-ovulation) at 4:00 pm were still 1-cell stage (E0.75) (data not shown). Next, to investigate the optimal condition of electroporation for effective introduction of CRISPR/Cas9 reagents into rat embryos, we examined tetramethylrhodamine-labelled dextran (3 kDa) solution as a fluorescent indicator to evaluate *i*-GOAND method. We instilled 2-2.5 μl solution containing tetramethylrhodamine-labelled dextran (0.5 μg/μl) into oviductal lumen of super-ovulated pregnant WKY female rats at E0.75 (Fig. 1a-c, Additional file 1: movie S1) and then electroporated them using a NEPA21 electroporator as described in the Methods section (Fig. 1d-f, Additional file 2: movie S2). We designed 5 different conditions of electroporation (voltage with poring pulse: 50 V, or 40 V, or 30 V; number of pulses with transfer pulse: 3 times or 6 times; see Fig. 1f, i) in WKY rat embryos. Two days after in vivo electroporation, the embryos were isolated from the treated females and examined presence/absence of fluorescence. In the embryos with successful delivery of the dextran, some embryos exhibited distinct tetramethylrhodamine-derived red fluorescence in their cytoplasm (Fig. 1g, h). As shown in Fig. 1i and Table 1, the 6 times of transfer pulse is highly efficient to the electroporation compared with those of 3 times. To investigate the differences of the electroporation efficiency in other rat stains, DA and WKY/DA F1 rat embryos were performed. In all 3 rat strains, there was no detectable difference in the delivery efficiency among stains (Fig. 1i-k, Table 1). These data suggest that “voltage with a poring pulse: 50V, and number of pulses with transfer pulse: 6 times” in the most suitable condition in rats. We termed this GONAD method as *R*at improved GONAD (rGONAD).

**Table 1:** Fluorescence ratio of teratmethlrhodamin-labelled dextran in rat embryos

Stain	Poring Pulse Voltage (V)	Transfer pulse No. of Pulses	No. of > 2-cell embryos (A)	No. of embryos with fluorescence (B)	% (B/A)
WKY	50	3	138	68	49.3
	50	6	116	86	74.1
	40	3	158	36	22.8
	40	6	124	56	45.2
	30	6	123	38	30.9
DA	50	6	118	88	74.6
	40	6	109	41	37.6
	30	6	110	23	20.9
WKY x DA (F1)	50	6	122	67	54.9
	40	6	154	54	35.1
	30	6	133	31	23.3


Additional file 1:Instillation of the solution containing tetramethylrhodamine-labelled dextran into oviductal lumen WKY female rats. (MP4 2741 kb)



Additional file 2:Electroporation using a NEPA21 electroporator. (MP4 6219 kb)


### rGONAD-based gene disruption (KO)

**Fig. 2:**
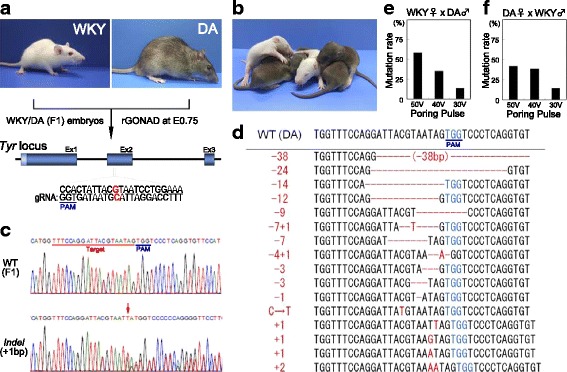
Production of *Tyr* knock-out (KO) rats by the rGONAD method. **a** Scheme of allele-specific genome editing for knock-out rats. The rGONAD method was performed in 0.75 day pregnant *albino* WKY crossed to *agouti* DA strains, which the fertilized eggs are (WKY x DA) F1 hybrid. The target sequence and PAM at *Tyr* locus are shown. **b** Some the editing rats had *albino* colored coats. **c** Direct sequencing results of wild-type F1 (upper; WT) or the editing (below; *indel*) rats. Red arrow indicates *indel* mutaion. **d** Sequence analysis of the pups showed a variety of *indel* mutation at the *Tyr* locus, as shown in red. **e**, **f** Graph shows analysis of the percentage of genome edited efficiency in *Tyr* gene in WKY female x DA male (**e**) and WKY female x male (**f**)

Then, to investigate whether rGONAD is feasible to generate genome-edited rats by delivery of CRISPR/Cas9 components, tyrosinase (*Tyr*) gene, encoding the enzyme responsible for the melanin synthesis, was used as a target of this experiment as previously reported [25]. The gRNA targeted to the wild-type *Tyr* allele was used to test the allele-specific genome-editing. Cas9 protein and gRNA were instilled into the oviducts of E0.75 WKY and DA female rats containing the fertilized eggs with hybrid genotypes (WKY x DA, F1), and electroporation was performed (Fig. 2a). The conditions of electroporation were the voltage with poring pulse: 50 V, or 40 V, or 30 V; number of pulses with transfer pulse: 6 times.

Unfortunately, we could hardly obtain pups from super-ovulated female rats in our initial trial (WKY: injected females; 12, pregnant females; 2; DA: injected females; 14, pregnant females; 0). Therefore, we decided to use the estrous female rats without super-ovulation for the following study. Such females became successfully pregnant and delivered pups (Table 2, Additional file 3: Table S1). Some pups showed *albino*-colored coat, indicating disruption of wild-type *Tyr* gene allele (Fig. 2b). Sequence analyses of their genomic DNA indicated that all the pups showing *albino* phenotype carried *indel* mutations at the target region of the *Tyr* gene (Fig. 2c, d). Of the pups that were electroporated with 50, 40, and 30 V of the voltage, 58.7, 35.3, and 13.4%, the pups had edited *Tyr* gene, respectively (Table 2, Fig. 2e, Additional file 3: Table S1). No significant difference was detected in the gene editing efficiency between the strains of female rats; WKY and DA (Table 2, Fig. 2f, Additional file 3: Table S2), suggesting that r-GONAD is adaptable for all rat strains.

**Table 2:** *Tyr*-mediated mutations in F1 (WKY x DA) rat

Stain	Poring Pulse Voltage (V)	Injected	Pregnant	Pups (A)	KO (B)	% (B/A)
WKY♀ x DA♂	50	10	9	46	27	58.7
	40	10	9	68	24	35.3
	30	9	8	67	9	13.4
DA♀ x WKY♂	50	9	6	19	8	42.1
	40	9	5	26	10	38.5
	30	7	6	28	4	14.3

In addition, to study possible germ-line transmission of mutated alleles in rats obtained by the rGONAD, we obtained several F1 offspring by crossing between F0 founders and wild type rats. The germline transmission was confirmed in the next generations (Additional file 3: Table S3). These results indicate that it is possible to create genome-edited rat lines by the rGONAD method.

### Recovery of coat-color mutation using ssODN-based knock-in (KI) approach

**Fig. 3:**
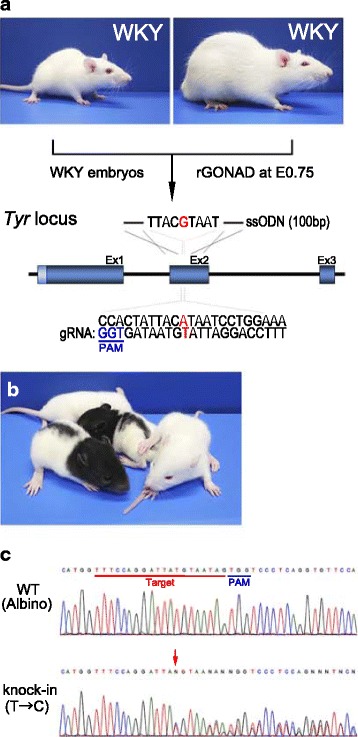
Recovery of coat-color mutation in *albino* WKY rats using KI approach. **a** Scheme diagram of the recovery of coat-color mutation. The rGONAD method was performed in 0.75 day pregnant WKY female rats crossed to WKY male rats. The target sequence, PAM, and ssODN at *Tyr* locus are shown. **b** Picture of the knock-in rats showing the *albino* with *non-agouti*, *hooded* phenotype. **c** Direct sequencing results of wild-type WKY (upper; WT) or the editing (below; Knock-in) rats. Red arrow indicates that one allele had the corrected sequence (T to C)

To analyze whether rGONAD method can be used to introduce genetic changes in rat genome, we next examined to repair mutation associated with representative recessive coat-color phenotypes in rat, as previous report [25]. To recover the *albino* phenotype caused by the point mutation, we designed a gRNA for a region spanning the mutation and a 100-bases long ssODN repair template that corresponds to the wild-type sequence of *Tyr* gene (Fig. 3a). These components were instilled into oviducts of pregnant WKY rats and subjected to electroporation. As a result, some of the pups showed *albino* with *non-agouti*, *hooded* phenotype, indicating successful correction of *Tyr* gene mutation (Fig. 3b). Sequence analyses of genomic DNA demonstrated that the pups showing pigmentation phenotype contained the corrected *Tyr* sequence (Fig. 3c). Of the pups that obtained from females that underwent electroporation with 50 and 40 V poring pulse, 50.0 and 17.8%, had *indel* mutation (knock-out; KO), and 26.9 and 11.1%, had corrected alleles (knock-in; KI) at the target region, respectively (Table 3, Additional file 3: Table S4). Thus, these observations suggest that the rGONAD method is obviously feasible for gene correction in rat by the ssODN-based knock-in.

**Table 3:** Coat-color phenotypes recovered from *albino* in WKY rat

Stain	Poring Pulse Voltage (V)	Injected	Pregnant	Pups (A)	KO (B) (%: B/A)	KI (C) (%: C/A)
WKY x WKY	50	8	6	26	13 (50)	7 (26.9)
	40	9	7	45	8 (17.8)	5 (11.1)

## Discussion

The present study showed that the most suitable conditions for rGONAD method confirmed by electroporation efficiency. The rGONAD method allows efficient production of gene disrupted rats using CRISPR/Cas9 system. Furthermore, we demonstrated high efficiency of rGONAD method to produce knock-out and knock-in rats.

CRISPR/Cas9 system can be provided in genetically modified animals. However, it is difficult for the researchers to require high level technical expertise such as micromanipulation. Recently, electroporation-mediated in vitro transfection of isolated pre-implantation embryos has been reported in rats (TAKE methods) [26, 27]. Although this method is very convenient, it is limited only to bypass the microinjection step; the other two steps, isolation of embryos and embryo transfer could not be excluded.

On the contrary, the rGONAD method certainly avoided all the 3 major steps of producing the genome-editing rats. Thus, these features suggested that the rGONAD method is more reliable that does not require any specific skills and equipment such as micromanipulator and inverted microscope.

In our study, we were not able to obtain genome-editing rats using the rGONAD method in the super-ovulated female rats whereas the manipulated female rats became pregnant. This problem occurred in the super-ovulated female rats might be due to the following possibilities. In previous reports, super-ovulated female rats mated to males tended to low pregnancy rates though the number of blastocyst implantation in the super-ovulated females did not differ from those in normal rats [28, 29]. Similarly, our reports indicated that there were few pups in the super-ovulation with WKY and DA rats. On the other hand, we successfully demonstrated the rat rGONAD method without super-ovulated females. Moreover, we described a protocol for high efficiency for genome-editing using rGONAD method as well as microinjection or in vitro electroporation. However, further studies are needed to draw a definite conclusion concerning super-ovulation and rGONAD method.

The laboratory rat has long been recognized as the preferred experimental animal in many areas of biomedical science [21]. In our previous study, anti-GBM nephritis can be induced in WKY rats by injection of monoclonal antibody against the NC1 domains of type IV collagen alpha chains [7, 8]. Although much progress has been made in understanding molecular mechanisms regulating anti-GBM nephritis, little is known about regarding molecular mechanism using gene disrupted rats.

In the present study, we showed high efficiency of the rGONAD method to produce knock-out and knock-in WKY rats. Taken together, using gene disrupted WKY rats as identified gene(s), the rat model of passively induced anti-GBM nephritis has tremendous potential for examining the mechanism by which inflammation is induced in human anti-GBM nephritis, as well as for identifying potential therapeutic targets. In addition in rats, there are several strains in order to perform specific studies, such as F344 for neurogenesis and SHR for hypertension [30]. In the future study, provided the genome-edited strain rats using the rGONAD method can be used in a variety of experiments.

In the recent study, it is certainly essential to meet with 3R principles of animal experimentation; Reduction, Replacement and Refinement, and further to understand welfare of animals [31]. The *i*-GONAD method does not require euthanasia of pregnant females, unlike in the traditional approaches where females need to be sacrificed for isolating zygotes for introduction of genome editing components ex vivo [24]. Moreover, we found that some female rats which underwent rGONAD method were able to deliver the pups (data not shown). These data indicate that the operated oviducts in the treated rats retained normal reproductive functions, pregnancy, and delivery. Finally, we strongly suggest that producing genome-editing animals with rGONAD method can be useful in variety of experimentation.

## Conclusions

In conclusion, we established the method, namely rGONAD for generating genome editing rats. We confirm that the rGONAD method will facilitate the production of rat genome engineering experiments in many laboratories. Furthermore, rGONAD method can be readily applicable where traditional gene targeting using ES cells will not be well established in mammals such as guinea pig, hamster, cow, pig, and other mammals.

## Methods

### Animals

WKY/NCrl rats were obtained from Charles River, and DA/Slc rats were purchased from SLC Japan. The rats were kept with a 12:12-h light: dark cycle.

To investigate the electroporation efficiency (Fig. 1), rat females (WKY and DA) were super-ovulated by intraperitoneal injection of 20 IU pregnant mare serum gonadotropin (PMSG; ASKA Pharmaceutical Co. Ltd., Tokyo, Japan) and 10 IU human chorionic gonadotropin (hCG; ASKA Pharmaceutical Co. Ltd.). The super-ovulated females were mated to rat males (WKY and DA) overnight. To analyze CRISPR/Cas9 systems (see Figs. 2 and 3), estrous female rats were mated to male rats without super-ovulation. Presence of copulation plugs was confirmed by visual inspection in the following morning and used for the electroporation experiments.

All animals were handled in strict accordance with good animal practice as defined by the relevant national and/or local animal welfare bodies, and all animal works were approved by the appropriate committee.

### Preparation of fluorescence-labelled dextran

To test the conditions of electroporation, we prepared 0.5 mg/ml Tetramethylrhodamine-labelled dextran (3 kDa; Thermo Fisher Scientific, Walttham, MA) and 0.1% trypan blue (Nacalai tesque, Kyoto, Japan) diluted in OPTI-MEM (Thermo Fisher Scientific).

### Preparation of CRISPR/Cas9 regents

For the preparation of CRISPR/Cas9 regents, Alt-R™ CRISPR-Cas9 system (Integrated DNA Technologies [IDT, Coralville, IA]) was used in accordance with the manufacturer’s protocol. Briefly, guide RNAs were designed using CHOPCHOP (http://chopchop.cbu.uib.no/). The synthetic crRNA and tracrRNA were commercially obtained as Alt-R™ CRISPR guide RNAs from IDT together with Cas9 protein (Alt-R™ S.p. Cas9 Nuclease 3NLS). Lyophilized crRNA and tracrRNA were resuspended in RNA-free Duplex Buffer to a concentration of 200 μM. Equal volume of crRNA and tracrRNA were mixed, and were heated at 95 °C for 5 min. Then the mixture were removed from heat and placed at room temperature (20-25 °C) for more than an hour before the electroporation. ssODN donor (Additional file 3: Table S5) was custom synthesized from Eurofins Genomics Japan (Tokyo, Japan).

### rGONAD method

*i*-GONAD method was performed as described previously [24]. Surgical procedures were operated on anesthetized female rats at Day 0.75 of pregnancy (E0.75: corresponding to late 1-cell stage zygotes; at 16:00 of the same day when plugs were confirmed) under observation using a dissecting microscope (SZX7; Olympus, Tokyo, Japan). Before injection, The annealed crRNA and tracrRNA were mixed with Cas9 (and ssODN) so that the final concentrations of components 30 μM (for crRNA/tracrRNA), 1 mg/ml (for Cas9 protein), 1 μg/μl (for ssODN) diluted in OPTI-MEM and placed at room temperature (20-25 °C) for 5 min. Approximately 2.-2.5 μl of electroporation solution (Tetramethylrhodamine-labelled dextran or CRISPR/Cas9 solution) was injected into the oviductal lumen from upstream of the ampulla using a micropipette. The micropipette apparatus consisted of a glass capillary needle (pulled using an electric puller: PN-31; Narishige, Tokyo, Japan). The electroporation was performed using a NEPA21 (NEPA GENE Co. Ltd., Chiba, Japan), generating two types of square pulses, namely poring and transfer pulses. The electroporation parameters were as follows; Poring pulse; 50 or 40 or 30 V, 5 msec pulse, 50 msec pulse interval, number of pulse 3 times, 10% decay (± pulse orientation) and Transfer pulse; 10 V, 50 msec pulse, 50 msec pulse, number of pulse 3 or 6 times, 40% decay (± pulse orientation) (see Fig. 1e, f). The rats were monitored for anesthesia recovery and were housed for further analysis.

### Genotyping

Gene alterations were certified by PCR followed by DNA sequencing, as described previously [32]. Briefly, rat genomic DNA was isolated from the ear-piece or tail. PCR amplification was performed using the following primers. Direct sequencing was performed using the PCR products. PCR and sequence primers with the following primers: Tyr-fw (5′- ttggttttcacagatcatttg-3′), Tyr-rv (5′- gctgaaattggcagttctatc-3′).

### Fluorescent signal detection

We collected the embryos at 2 days after electroporation. The embryos were observed using the fluorescence inverted microscope (Olympus IX73) for detecting the tetramethylrhodamine fluorescence.

## Additional files

Additional file 3: **Table S1.** Tyr-mediated mutations in F1 (DA male/WKY female) rat. **Table S2.** Tyr-mediated mutations in F1 (WKY male/DA female) rat. **Table S3.** Tyr-mediated mutations in F1 offspring. **Table S4.** Coat-color phenotypes recovered from *albino* in WKY rat. **Table S5.** CRISPR/Cas9 target sequence and ssODN used. (PDF 46 kb)

## Abbreviations

Cas: CRISPR-associated
CRISPR: Clustered Regularly Interspaced Short Palindromic Repeats
GONAD: Genome editing via Oviductal Nucleic Acids Delivery
hCG: Human chorionic gonadotropin
*i*-GONAD: *i*mproved GONAD
PMSG: Pregnant mare serum gonadotropin
rGONAD: Rat improved GONAD
ssODN: Single stranded oligonucleotides
TALEN: Transcription activator-like effector nucleases
Tyr: Tyrosinase
WKY: Wistar Kyoto
ZFN: Zinc finger nucleases
