# Supplementary material for: Successful production of genome-edited rats by the rGONAD method
Source: BMC Biotechnol. 2018 Apr 2;18:19. doi: 10.1186/s12896-018-0430-5 (PMC5879918; doi:10.1186/s12896-018-0430-5)
Supplement: Supplementary file 3 — Table S1. Tyr-mediated mutations in F1 (DA male/WKY female) rat. Table S2. Tyr-mediated mutations in F1 (WKY male/DA female) rat. Table S3. Tyr-mediated mutations in F1 offspring. Table S4. Coat-color phenotypes recovered from albino in WKY rat. Table S5. CRISPR/Cas9 target sequence and ssODN used. (PDF 46 kb) [file 12896_2018_430_MOESM3_ESM.pdf]

Poring Pulse Voltage (V)	Transfer pulse No. of Pulses	G0 newborns	KO
50	6	8	3
50	6	10	5
50	6	3	3
50	6	4	3
50	6	0	
50	6	5	3
50	6	2	1
50	6	10	8
50	6	2	0
50	6	2	1
	total	46	27

Poring Pulse Voltage (V)	Transfer pulse No. of Pulses	G0 newborns	KO
40	6	10	7
40	6	12	5
40	6	0	
40	6	5	3
40	6	3	0
40	6	7	3
40	6	9	1
40	6	6	1
40	6	10	4
40	6	6	0
	total	68	24

Poring Pulse Voltage (V)	Transfer pulse No. of Pulses	G0 newborns	KO
30	6	10	0
30	6	10	0
30	6	11	6
30	6	10	0
30	6	0	
30	6	3	1
30	6	5	1
30	6	12	0
30	6	6	1
	total	67	9

Table S1. Tyr-mediated mutations in F1 (DA male/WKY female) rat

Poring Pulse Voltage (V)	Transfer pulse No. of Pulses	G0 newborns	KO
50	6	2	1
50	6	2	2
50	6	3	2
50	6	4	1
50	6	4	1
50	6	0	
50	6	0	
50	6	0	
50	6	4	1
	total	19	8

Poring Pulse Voltage (V)	Transfer pulse No. of Pulses	G0 newborns	KO
40	6	5	1
40	6	4	4
40	6	4	1
40	6	0	
40	6	8	2
40	6	0	
40	6	5	2
40	6	0	
40	6	0	
	total	26	10

Poring Pulse Voltage (V)	Transfer pulse No. of Pulses	G0 newborns	KO
30	6	0	
30	6	4	3
30	6	4	0
30	6	4	0
30	6	5	0
30	6	5	0
30	6	6	1
	total	28	4

Table S2. Tyr-mediated mutations in F1 (WKY male/DA female) rat

Poring Pulse Voltage (V)	F0 KO	F1 pups	F1 KO
50	female	13	10
	male	11	6
	male	13	8
	male	10	5
	female	10	1
40	female	12	6
	male	8	6
	female	12	6
	female	11	5
30	female	10	8
	female	10	4
	male	13	12

Table S3. Tyr-mediated mutations in F1 offspring

Poring Pulse Voltage (V)	Transfer pulse No. of Pulses	G0 newborns	KO	KI
50	6	1	0	1
50	6	0		
50	6	5	2	1
50	6	7	1	3
50	6	5	5	0
50	6	1	1	0
50	6	7	4	2
50	6	0		
	total	26	13	7

Poring Pulse Voltage (V)	Transfer pulse No. of Pulses	G0 newborns	KO	KI
40	6	10	3	1
40	6	3	1	0
40	6	0		
40	6	7	0	0
40	6	2	1	0
40	6	11	0	2
40	6	7	1	0
40	6	5	2	2
40	6	0		
	total	45	8	5

Table S4. Coat-color phenotypes recovered from *albino* in WKY rat

	Sequence	Figure used
gRNA	TTTCCAGGATTACGTAATAGTGG	Figure 2
gRNA	TTTCCAGGATTATGTAATAGTGG	Figure 3
ssODN	CTGAAGATGGGAGCCTGGGGGTTTT<br>GGCTTTGTCATGGTTTCCAGGATTA<br>CGTAATAGTGGTCCCTCAGGTGTTC<br>CATCACATAAAACCTGATGGCTATT	Figure 3

Table S5. CRISPR/Cas9 target sequence and ssODN used
